# Gender-Dependent Effect of Progesterone on the Expression of Metallothionein Genes in Rat Inguinal Adipose Tissue

**DOI:** 10.3390/ijms26094066

**Published:** 2025-04-25

**Authors:** Sylwia Szrok-Jurga, Jacek Turyn, Julian Swierczynski, Wiktoria Stelmanska, Malgorzata Presler, Ewa Stelmanska

**Affiliations:** 1 Department of Biochemistry, Faculty of Medicine, Medical University of Gdansk, 80-211 Gdansk, Poland; jacek.turyn@gumed.edu.pl (J.T.); malgorzata.presler@gumed.edu.pl (M.P.); 2 Institute of Nursing and Medical Rescue, State University of Applied Sciences in Koszalin, 75-582 Koszalin, Poland; juls@gumed.edu.pl; 3 Provincial Integrated Hospital in Elblag, 82-300 Elblag, Poland; stelmanska.w@gmail.com

**Keywords:** progesterone, metallothionein, adipose tissue, gene expression

## Abstract

Metallothioneins (MTs) are low-molecular-weight metal-binding proteins potentially involved in the detoxification of heavy metals, protection against oxidative stress, and other biological processes. This study examined progesterone’s influence on *Mt* gene expression in rat adipose tissue. Wistar rats (females and males) received 100 mg of progesterone per rat. MT mRNA and protein levels were quantified by real-time PCR and Western blotting methods. Using radioimmunoassay, the serum progesterone level was measured. In this study, progesterone administration to female rats led to a 2.5-fold increase in serum progesterone concentration and significant increases in MT-1, MT-2A mRNA, and protein levels in inguinal WAT (WATi), compared to untreated female rats. RU 486 (progesterone receptor antagonist) abolished progesterone’s influence on *Mt-1* and *Mt-2A* gene expression in female WATi. Progesterone administration did not alter the level of *Mt-3* gene expression in WATi or *Mt-1* and *Mt-2A* in retroperitoneal WAT or brown adipose tissue in female rats.

## 1. Introduction

Metallothioneins (MTs) are low-molecular-weight (6–7 kDa) metal-binding proteins [1,2] that have many essential biological functions [3,4,5]. MTs are significantly involved in the control and detoxification of heavy metals. Moreover, they have a protective role in oxidative stress, scavenging free radicals [6]. In humans and rodents, MTs are conventionally divided into four groups [7,8,9]. In rodents, four functional *Mt* genes are known: *Mt-1*, *Mt-2A*, *Mt-3*, and *Mt-4* [4]. The *Mt-1* and *Mt-2A* isoforms are expressed in many cell types of most organs. Those isoforms are mostly found in the liver, kidney, intestine, and pancreas [2]. *Mt-3* is expressed predominantly in neurons, glia, and male reproductive organs [10] while *Mt-4* appears to be expressed exclusively in stratified squamous epithelial cells [11]. Immunohistochemical studies have demonstrated increased MT levels in the cytoplasm and nuclei of rapidly proliferating cells [4]. Relatively little is known about the biological functions of MT-3 and MT-4.

The expression of the *Mts* gene can be induced by many stimuli, including heavy metals, interferon, interleukin-1, vitamin D3, endotoxins, and steroid hormones such as glucocorticoids and progestins [4,6]. Ljubojević et al. [12] found that the expression of *Mt*-*1* and *Mt-2A* genes in the liver and kidney was female-dominant, up-regulated by castration, and down-regulated by ovariectomy. Progesterone also increased the *Mt-1* gene expression in a rat liver cell line [13]. In human cell lines containing functional progesterone receptors, *MT-2A* gene expression was induced by synthetic progestins (R5020 and medroxyprogesterone acetate) [14].

It should be noted that MT-1 mRNA expression begins to increase in the ovary shortly after ovulation, when increased progesterone production is observed [15]. Other studies indicate that progesterone decreases MT-1 mRNA levels in the livers of ovariectomized mice [16]. Based on the data presented above, one can assume that progesterone is also one of the factors that may affect the *Mts* gene expression in some organs, especially in the liver and kidneys.

Some data have indicated that *Mt* genes are expressed in white (WAT) and brown adipose tissue (BAT) [17,18,19]. Trayhurn et al. [18,20] suggested that MTs belong to adipokines, cell-signaling proteins synthesized and secreted by adipose tissue that regulate diverse processes, including lipid storage (obesity), inflammation, autoimmunity, insulin secretion, and reproduction [21,22]. Murphy et al. revealed that progesterone specifically enhances the expression of the *MTs* gene family in the epithelial cells of endometrial organoids, while elevated adiposity significantly reduces the expression of the *MT* genes. The repression of *MT* genes results in increased DNA damage, indicating a protective role for *MT* genes [23]. Our previous studies [24,25,26] demonstrated that progesterone influences lipid metabolism and some adipokine production in the WAT of female rats. Considering that there is no information about the effects of progesterone on *Mts* gene expression in adipose tissue, and the suggestion that MTs might play an anti-obesity role, we hypothesize that progesterone may affect *Mts* gene expression in the WAT and BAT of rats.

The aim of the present study was to examine the effects of progesterone administration on *Mts* gene expression in different depots of adipose tissues of female and male rats.

## 2. Results

Given that *Mt-1* and *Mt-2A* gene expression are affected by the amounts of food consumed by male rats [19], first, we determined the food intake of the male and female rats, treated and not treated with progesterone. The composition of the diet is included in the Supplementary Materials (Table S1). The results described in Table 1 indicate that the amount of food consumed by progesterone-treated females was significantly increased compared to the control female rats. Moreover, there was no difference in food consumption between the male groups (progesterone-treated and control).

**Figure 1:**
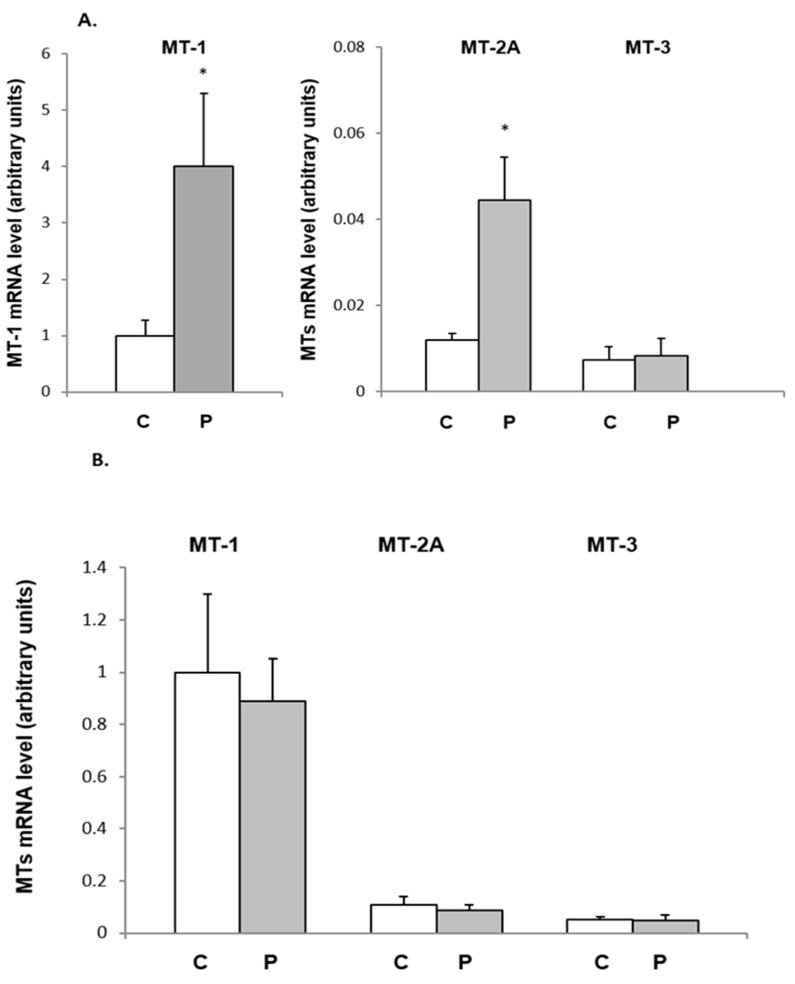
At the top, the effect of progesterone administration on *Mt1* gene expression in the inguinal WAT (WATi) of female rats (**A**). The MT-1 mRNA level relative to β-actin expression in the WATi of control (C) and progesterone-treated (P) females is shown on the graph (mean ± SEM, *n* = 10). * *p* < 0.05 control females versus progesterone-treated females. The MT-2 and MT-3 mRNA levels relative to β-actin gene expression in the WATi of control (C) and progesterone-treated (P) females are shown on the graph (mean ± SEM, *n* = 10). Results are compared to the *Mt1* gene relative expression (right panel). At the bottom, the influence of progesterone on MTs mRNA level in the inguinal adipose tissue of males (**B**). MTs mRNA level relative to β-actin expression in the inguinal adipose tissue of control (C) and progesterone-treated (P) groups is shown on the graph (mean ± SEM, *n* = 10). Results are described in comparison to the relative expression of the *Mt1* gene.

The results described in Figure 1A indicate that the *Mt-1*, *Mt-2A*, and *Mt-3* genes are expressed in the inguinal WAT (WATi) of female rats. Using the same method (real-time PCR), no MT-4 mRNA was detected in this WAT depot. Administration of progesterone to female rats caused an approximately 4-fold increase in MT-1 and MT-2A mRNA levels in the WATi. No effect of progesterone administration on MT-3 mRNA was observed (Figure 1A). The MT-1 mRNA level was several times higher than the MT-2A and MT-3 mRNA levels, respectively (Figure 1A, white bars). Thus, these results indicate that *Mt-1* is expressed in the most significant amounts in the WATi of female rats.

Similarly to the results associated with females, MT-1, MT-2A, and MT-3 mRNA were also detected in male rats’ inguinal adipose tissue (Figure 1B). Again, *Mt-1* was expressed in the most significant amounts in the WATi of male rats (Figure 1B, white bars). Similarly to the results for females, no MT-4 mRNA was detected in male rats’ inguinal adipose tissue. In contrast to the results for female rats, no effect of progesterone administration on *Mt-1* and *Mt-2A* gene expression in the WATi of male rats was observed (Figure 1B, grey bars).

**Figure 2:**
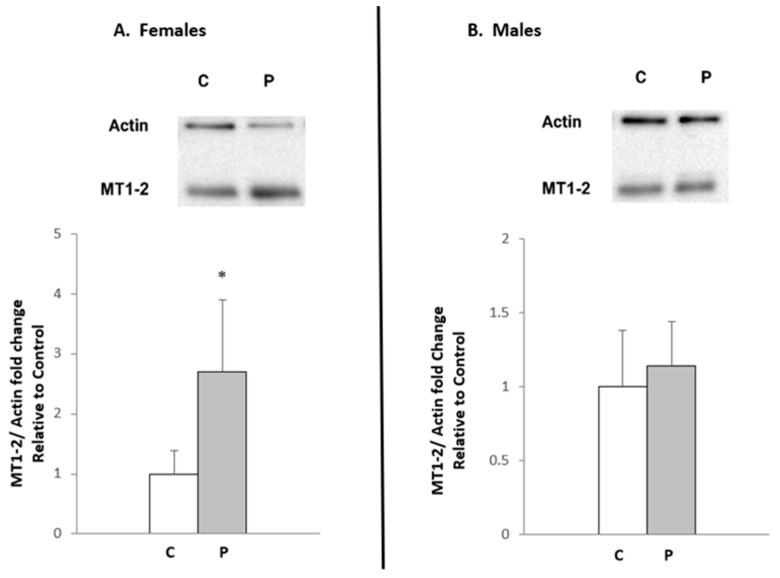
The effect of progesterone administration on MT1-2 protein levels in the inguinal WAT of female (**A**) and male (**B**) rats. C: control rats (*n* = 5), P: progesterone-treated rats (*n* = 5). MT protein levels were assessed by Western blotting (one representative immunoblot is shown) with 20 μg of protein (whole tissue homogenate) per lane. Actin was used as a standard. The molecular mass of MT1-2 was estimated by comparing it to the molecular mass of protein markers. One band corresponds to two different isoforms of MT (MT-1 and MT-2A). The graphs represent a densitometric analysis of the above Western blot results. Western blots were normalized to actin, and densitometric analysis was performed using Image Lab 5.1 (Bio-Rad, Hercules, CA, USA). Values are mean ± SEM of five experiments. * *p* < 0.05.

As expected, Western blot analysis revealed a significant increase in the MT protein level in the WATi of progesterone-treated female rats, compared to controls (Figure 2A). It should be mentioned that the antibody used in this experiment recognizes both the MT-1 and MT-2A isoforms. Thus, the results presented in Figure 2A further confirm the hypothesis that progesterone is an essential factor stimulating both *Mt-1* and *Mt-2* gene expression in the WATi of female rats. As expected, no significant differences in WAT MTs protein levels in progesterone-treated male rats, versus controls, were observed (Figure 2B). Moreover, densitometry analysis of Western blots confirmed statistically significant increases in MT-1 and MT-2A levels after progesterone administration, in comparison to control (almost 3-fold) in females’ WATi and no hormonal effect on MTs protein levels in males (Figure 2A,B).

To verify whether the observed increases in *Mt-1* and *Mt-2A* gene expression in the WATi of female rats are the direct effect of progesterone, we measured its concentration in the serum of control and progesterone-treated rats. Administration of a single dose of progesterone in female rats resulted in an increase of the serum hormone concentration from 31.3 ± 6.7 ng/mL (value observed in female control rats) to 76.8 ± 8.4 ng/mL (value observed in progesterone-treated female rats; *p* < 0.01). This indicates the direct link between increased *Mt-1* and *Mt-2A* gene expression in WATi and increased serum progesterone concentration.

Based on the data presented in Figure 1A and the serum progesterone concentration, the correlation coefficient between progesterone concentration and the MTs mRNA levels was calculated. A strong positive correlation was revealed between the MT-1 mRNA level in WATi and serum progesterone concentrations (correlation coefficient R = 0.80; *p* < 0.01). A similar correlation between MT-2A mRNA and serum progesterone concentration was found (correlation coefficient R = 0.83; *p* < 0.01).

**Figure 3:**
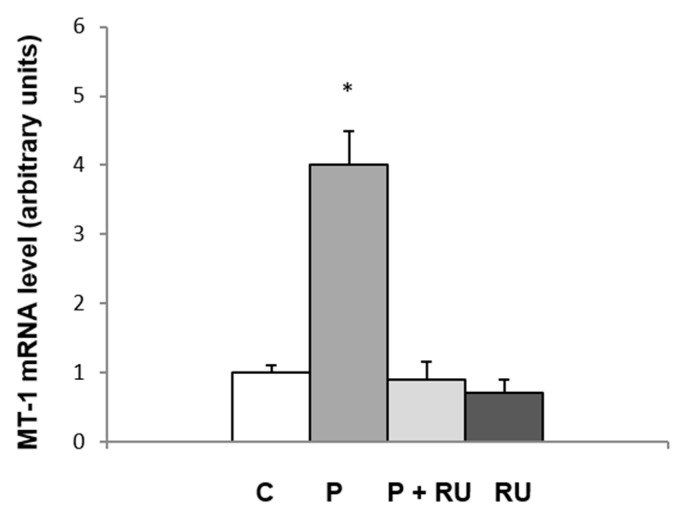
The influence of mifepristone (RU 486) on MT-1 mRNA level in the inguinal WAT of females. MT-1 mRNA levels relative to β-actin expression in the inguinal adipose tissue of control (C), progesterone-treated (P), progesterone and mifepristone-treated (P + RU), and mifepristone-treated (RU) females are shown on the graph (mean ± SEM, *n* = 10). * *p* < 0.05 progesterone-treated females versus remaining groups.

To further confirm that the increases in *Mt-1* and *Mt-2A* gene expression are directly linked to progesterone binding to a specific receptor, we administered progesterone together with mifepristone (also known as RU 486), a specific antagonist of the progesterone receptor, to the animals. The data presented in Figure 3 indicate that RU 486 completely abolishes the effect of progesterone on MT-1 mRNA levels in the WATi of female rats. Thus, the results presented in Figure 3 indicate that the effect of progesterone on *Mt-1* and *Mt-2A* gene expression in the WATi of female rats is directly linked to the hormone binding to the specific progesterone receptor.

It should be noted that the serum concentration of progesterone in control male rats (1.8 ± 0.5 ng/mL) was several times lower than in control female rats (31.3 ± 6.7 ng/mL). Administration of a single dose of progesterone in male rats resulted in an increase of the hormone concentration to 29.2 ± 7.3 ng/mL (male rats treated with progesterone vs. control males, *p* < 0.01). This means that the administration of progesterone in male rats increased the hormone concentration to the value observed in female control rats (see above).

**Figure 4:**
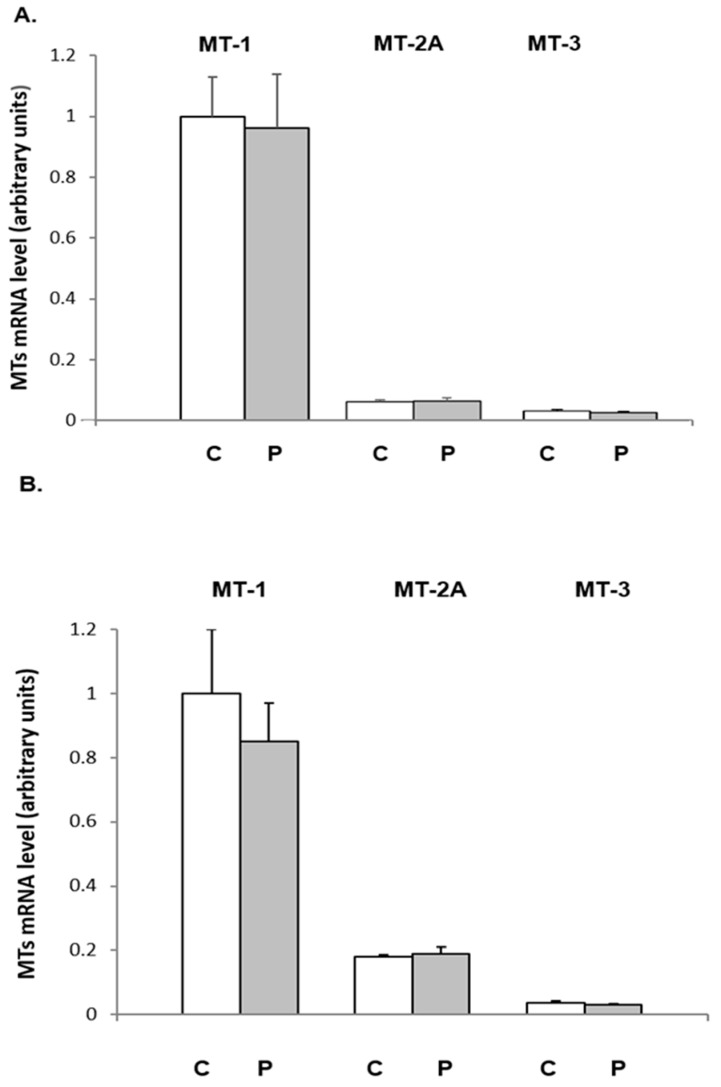
The influence of progesterone on MTs mRNA level in the retroperitoneal WAT (**A**) and brown adipose tissue (BAT) (**B**) of females. MTs mRNA levels relative to β-actin expression in the tested tissue of control (C) and progesterone-treated (P) groups are shown on the graph (mean ± SEM, *n* = 10). Results are described in comparison to the relative expression of the *Mt1* gene.

Finally, to obtain more information about the fat-depot specificity of progesterone action, we also examined the effects of progesterone administration on the MTs mRNA levels in the retroperitoneal WAT (Figure 4A) and interscapular BAT of female rats (Figure 4B). Relative to the WATi, *Mt-1* was expressed in more significant amounts in the retroperitoneal WAT and BAT of female rats. However, in contrast to inguinal adipose tissue, progesterone administration exerted no effect on MTs mRNA levels in the retroperitoneal WAT and BAT of females (Figure 4).

These data suggest that the stimulatory effect of progesterone on *Mt-1* and *Mt-2A* gene expression in the WAT of female rats is limited to the WATi.

## 3. Discussion

The most important findings of the present study are the gender-specific and WAT depot-specific effects of progesterone on *Mt-1* and *Mt-2A* gene expression. The results presented in this paper indicate, for the first time, that white adipose tissue *Mt-1* and *Mt-2A* gene expression are stimulated only in the WATi of female rats. No effect of progesterone on *Mt-1* and *Mt-2A* gene expression was found in the other WAT depots of female rats. Moreover, no effect of progesterone on *Mt-1* and *Mt-2A* gene expression was found in the inguinal (or other) WAT depots of male rats.

A strong correlation between serum progesterone concentrations and MT-1 and MT-2A mRNA levels in the WATi of female rats suggests that the effect of progesterone on *Mt-1* and *Mt-2A* gene expression in the WATi of female rats is directly linked to the hormonal action. Accordingly, the impacts of progesterone on *Mt-1* and *Mt-2A* gene expression were completely abolished by specific antagonists of progesterone receptors (RU 486). This suggests that progesterone stimulation of *Mt-1* and *Mt-2A* gene expression depends on the hormone binding to specific progesterone receptors in female rats’ WATi. It implies the specificity of progesterone-related action relating to *Mt-1* and *Mt-2A* gene expression in the WATi of female rats.

It has been reported that partially purified progesterone receptor binds to the promoter region of the *Mt-2A* gene in vitro [14]. Therefore, we can assume that in the WATi of female rats, progesterone is translocated into the nucleus and binds to the progesterone receptor. Then, the complex of progesterone and progesterone receptors as homodimers binds to the cis-acting progesterone response element present in the *Mt-1* or *Mt-2A* genes. These events result in an increase in *Mt-1* or *Mt-2A* gene transcription.

The results presented in this paper indicate that the amount of food consumed by progesterone-treated females was higher than that of the control female rats, whereas there was no difference in food consumption between male progesterone-treated and control rats. Thus, one can hypothesize that increases in *Mt-1* and *Mt-2A* gene expression could result from the amount of food consumed by progesterone-treated female rats. Szrok and others [19] reported that increases in *Mt-1* and *Mt-2A* gene expression are increased by fasting, while refeeding leads to decreases in *Mt-1* and *Mt-2A* gene expression. These results suggest that the up-regulation of *Mt-1* and *Mt-2A* gene expression by progesterone could be independent of the amounts of food consumed.

Based on the data presented in this paper, it is difficult to explain the lack of influence of progesterone on *Mt-3* gene expression. Similarly, it was reported that *Mt-3* in WAT is not regulated by fasting or fasting/refeeding in male rats, while *Mt-1* and *Mt-2A* significantly increased under these conditions [19]. Thus, one can assume that neither progesterone nor the amount of food consumed regulates *Mt-3* gene expression in WAT.

Previously, Stelmanska et al. [24] demonstrated a deficient level of progesterone receptors in the WATi of male rats. Therefore, the lack of progesterone-related action on *Mt-1* and *Mt-2A* gene expression in the WATi of male rats is probably associated with a lower level of progesterone receptor in the WATi of males, compared to female rats. In the present study, we found much lower serum progesterone concentration in males than in females. It should be noted that after the administration of progesterone to male rats, serum progesterone concentration reached the level (value) observed in female control rats. Therefore, it seems very likely that the much lower serum progesterone concentrations in male rats could at least partly explain why the MT-1 mRNA level was significantly higher in the WATi of control females compared to control males. The results presented in this paper suggest that the lack of effect of progesterone on *Mts* expression in male rats is probably caused by a very low level of progesterone receptors in the WATi [24] and lower serum progesterone concentration in male rats. Finally, the question arises: What is the physiological significance of the results presented in this paper? To answer that question, one has to consider three crucial problems: (a) serum progesterone concentration, (b) the potential physiological role of MTs, and (c) the effect of progesterone on human adipose tissue. It is well known that a significant increase in serum progesterone concentration (as in the data presented in this paper) is observed during pregnancy [27]. Thus, as far as serum progesterone concentration is concerned, such increases in the hormone are achievable in physiological conditions.

However, relatively little is known about the physiological role (especially in humans) of MT-1 and MT-2A. As mentioned (see the Introduction), MTs may be essential in detoxifying heavy metals and scavenging free radicals [6]. Assuming that progesterone stimulates MTs in human adipose tissue, it is very likely that MT-1 and MT-2A induction in WAT, exerted by progesterone during pregnancy, could protect the woman, and especially the developing fetus, from the toxic effects of heavy metals and play an antioxidant role, decreasing oxidative damage [18,28]. The protective effects of MT-1 and MT-2A against the toxicity of heavy metals or oxidative stress may also occur under other physiological and pathological conditions associated with elevated serum progesterone concentration and, consequently, with elevated levels of MT-1 and MT-2A. Accordingly, it was shown that a substantial increase in *Mt-1* gene expression might be necessary to protect the ovarian tissues from the oxidative stress generated by ovarian inflammatory events during the ovulatory process and luteinization [15].

In vivo experiments with transgenic animals demonstrated that mice lacking MT-1 and MT-2A become obese. Therefore, it was proposed that MT prevents obesity [29,30]. Moreover, studies performed on MT-lacking and MT-overexpressing female mice revealed that MTs protect the organism against high-fat-diet-induced obesity and associated effects such as insulin resistance, oxidative stress, and mitochondrial damage [18,30,31]. However, the assumption that MTs protect the organism against obesity still raises some controversy. For instance, human studies demonstrated that the level of MT-2A mRNA (a major metallothionein isoform in humans) is increased in the WAT of obese patients [31], which suggests that MT-2A could be a pro-obesity factor. Moreover, the data presented in this paper revealed that the *Mt-1*, *Mt-2A*, and *Mt-3* genes are expressed in the inguinal, retroperitoneal WAT, and interscapular BAT of females and male rats. We have also confirmed that *Mt-1* is expressed in the most significant amounts among the MT isoforms in the examined WAT and BAT depots of female and male rats. Northern blot analysis revealed previously that both the *Mt-1* and *Mt-2A* genes are expressed in each of the main fat depots of mice [32]. However, in contrast to our findings, no difference between the MT-1 and MT-2A mRNA levels in the WAT of mice was found, indicating that the level of expression of this gene is not depot-specific. Initially, using the Northern blot analysis, MT-1 mRNA was not detected in rat WAT [32]. However, the presence of MT-1 mRNA was demonstrated using real-time PCR [32]. These discrepancies between Trayhurn et al. [32,33] and our results could be explained by the different methods used to study MT mRNA levels. MT-3 mRNA was also detected in human (obese) subcutaneous and omental adipose tissue [34]. Previously, we detected MT-1, MT-2A, and MT-3 mRNA in the epididymal and retroperitoneal depots and the WATi of male rats [19]. Moreover, we demonstrated that genes encoding MT-1 and MT-2A, but not MT-3, in the retroperitoneal and epididymal depots and the WATi of male rats are regulated by nutritional status [19]. Moreover, we found that insulin plays an essential role in the regulation of *Mt-1* and *Mt-2A* gene expression in the retroperitoneal and epididymal depots and the WATi of male rats [19].

It has been demonstrated previously that the *Mt-1* and *Mt-2A* genes are expressed in rat adipocytes of brown fat [17]. To our knowledge, we have shown for the first time that the *Mt3* gene is expressed in the females’ BAT. According to Rodriguez et al., progesterone has been reported to lead to altered gene expression in BAT (e.g., induction of uncoupling protein 1—UCP1 mRNA expression) [35]. Our study revealed no effects of progesterone on the *Mt* gene expression in the BAT of females.

In summary, our findings imply that progesterone, by binding to a specific progesterone receptor, stimulates *Mt-1* and *Mt-2A* gene expression in the WATi of female rats. No effect of progesterone on *Mt-1* and *Mt-2A* gene expression was observed in the WATi of male rats. Our in vivo results seem to support the hypothesis that progesterone may exert beneficial effects during pregnancy, the ovulatory process, and luteinization via increased MT-1 and MT-2A levels. Interestingly, the stimulatory effect of progesterone on MT-1 and MT-2A mRNA and protein levels is limited to the WATi of female rats.

## 4. Materials and Methods

### 4.1. The Animals and the Treatment

- progesterone administration

Ten-week-old female and male Wistar rats were used, initially weighing approximately 200 g and 260 g, respectively. The rats were assigned to 4 groups: control females (*n* = 10), females treated with progesterone (*n* = 10), control males (*n* = 10), and males treated with progesterone (*n* = 10). Under general anesthesia induced by intraperitoneal injection of ketamine (60 mg/kg) and xylazine (6 mg/kg), a pharmacological dose (100 mg per rat) of progesterone (Sigma–Aldrich, St. Louis, MO, USA) was implanted subcutaneously in the lower abdomen. Control animals underwent an identical sham operation. The rats treated with progesterone and control rats (not treated with the hormone) were housed in individual wire-mesh cages in the same room at 22 °C, under a light/dark (12:12 h) cycle with lights on at 7:00 a.m. The animals were allowed free access to tap water and food (previously described commercial diet composition) [36]. The amount of food consumed was measured every other day. The animals were killed by decapitation 28 days after progesterone implantation. Adipose tissues (WAT and BAT) were collected, rapidly frozen in liquid nitrogen, and stored at −80 °C for the subsequent analyses. (Inguinal adipose tissue was removed bilaterally from the area located in the posterior region that extends from the dorsolumbar area to the gluteal region. All procedures were conducted manually with scissors and tweezers according to the protocol described in our previous manuscript [37]. Blood was collected from the neck artery, centrifuged, and the separated serum was stored at −80 °C until the progesterone concentration was measured.

- RU 486 administration

Two weeks after progesterone implantation, another two groups of female rats (control and progesterone treatment, *n* = 10 each) were treated with RU 486 (Molekula Limited, Dorset, UK). RU 486 (90 mg per rat) was implanted subcutaneously in the lower abdomen. The animals were treated as described above.

### 4.2. RNA Isolation

Total cellular RNA was extracted from the frozen tissues using the Purezol reagent containing guanidine thiocyanate and phenol (Bio-Rad, Hercules, CA, USA). One ml of Purezol was added to 100 mg of tissue and homogenized. After 5 min at room temperature, samples were centrifuged at 12,000× *g* for 10 min at 4 °C. Subsequently, the upper lipid layer was eliminated as the extract was transferred to new tubes. A quantum of 0.2 mL of chloroform was added to each sample. The contents of the tubes were thoroughly mixed. Afterward, samples were centrifuged at 12,000× *g* for 15 min at 4 °C. The centrifugation process separated the mixture into three distinct phases: an upper aqueous phase, an intermediate phase, and a lower organic phase. The aqueous phase, containing RNA, was subsequently transferred to new 2 mL tubes. To precipitate RNA, 0.5 mL of isopropyl alcohol was added to each sample, mixed, and after 5 min, centrifuged at 12,000× *g* for 10 min at 4 °C. Following the precise removal of the supernatant, the RNA within the precipitate was purified by adding 1 mL of 75% ethanol. The samples were centrifuged at 7500× *g* for 5 min at 4 °C. The supernatant was removed, and the precipitate was dried for 5 min at room temperature. The RNA was dissolved in 50 µL of water free of RNA-degrading enzyme activity (treated with DMPC). The RNA concentration was determined from the absorbance at 260 nm. All samples had a 260/280 nm absorbance ratio of approximately 2.0.

### 4.3. cDNA Synthesis

First-strand cDNA was synthesized from 4 μg total RNA (RevertAidTM First Strand cDNA Synthesis Kit; Thermo Fisher Scientific Inc., Lenexa, KS, USA). Before the amplification of cDNA, each RNA sample was treated with RNase-free DNase I (Thermo Fisher Scientific Inc., Lenexa, KS, USA) at 37 °C for 30 min. Determination of MT-1, MT-2A, MT-3, and MT-4 mRNA levels was accomplished by real-time PCR.

MT-1, MT-2A, MT-3, and MT-4 mRNA levels were quantified by real-time PCR using Chromo4 Real-Time Detection System (Bio-Rad Laboratories, Inc., Hercules, CA, USA). The primers were designed using BLAST-Primer 3 (version 2.5.0) (https://www.ncbi.nlm.nih.gov/tools/primer-blast/ accessed on 1 February 2025) (NCBI, Bethesda, MD, USA), from gene sequences obtained from the Nucleotide database (https://www.ncbi.nlm.nih.gov/nucleotide/ accessed on 1 February 2025) and synthesized at Genomed (Warsaw, Poland). The following oligonucleotide primers pairs were used: 5′-TGTCACCAACTGACGATA-3′ (forward) and 5′-GGGGTGTTGAAGGTCTCAAA-3′ (reverse) for β-actin (Actb); 5′-CCCGTGGGCTGCTCCAAATGT-3′ (forward) and 5′-ACTGGGTGGAGGTGTACGGCA-3′ (reverse) for MT-1; 5′-GCGATCTCTCGTTGATCTCC-3′ (forward) and 5′-CAGGAGCAGGATCCATCTGT-3′ (reverse) for MT-2A; 5′-TGGTTCCTGCACCTGCTCGG-3′ (forward) and 5′-TGGGAGTCCTCACTGGCAGCA-3′ (reverse) for MT3; and 5′-ACACACCTGGACCATGGACCCT-3′ (forward) and 5′-AGCAGGGGCAGCAGCTTTTACG-3′ (reverse) for MT4.

Real-time PCR amplification was performed as described previously [38]. The quantification of β-actin mRNA was performed on the corresponding samples. Subsequently, the results were normalized to account for any variations in the amount of RNA added and the efficiency of the reverse transcription. Relative quantities of transcripts were calculated using the 2^−ΔCT^ formula [39]. The results are expressed in arbitrary units, with one unit being the mean mRNA level determined in the control group. Amplification of specific transcripts was further confirmed by obtaining the melting curve profiles and subjecting the amplification products to 1% agarose gel electrophoresis.

### 4.4. Western Blot Analysis of Metallothionein

The Western blot procedure described previously was used to evaluate the relative abundance of MT protein [40,41]. Briefly, aliquots of the homogenates containing 10 µg protein were separated by 12% SDS-PAGE and electroblotted to Immun-Blot TM PVDF Membrane (Bio-RAD Laboratories, Hercules, CA, USA). The membrane containing the proteins was blocked for two hours in 5% BSA dissolved in 1 × PBS + 0.05% Tween 20. Next, it was incubated for 24 h at 4 °C with primary monoclonal mouse antibodies UC1MT against MT1 and MT2 (Abcam, Cambridge, UK) (antibody dilution 1:1000). After the indicated time, the membrane was washed three times for ten minutes in buffer 1 × PBS + 0.05% Tween 20. Afterwards, the membrane was incubated with secondary rabbit anti-mouse IgG antibodies conjugated to horseradish peroxidase (dilution 1:5000) (1 h at room temperature). In the next step, the membrane was again washed three times. The image was obtained by the chemiluminescence method using the ECL Western Blotting Substrate Kit (ChemiDoc™ XRS Apparatus; Bio-Rad). Actin levels were also determined in an analogous manner to standardize the results. The membrane was incubated for one hour with polyclonal rabbit antibodies against actin (1:400 dilution). After washing (three times), the membrane was incubated for one hour with anti-rabbit IgG and HRP-conjugated secondary antibodies (dilution 1:5000), rewashed, and relative protein levels were determined by chemiluminescence. Polyclonal rabbit antibodies against actin (A5060) and HRP-conjugated secondary antibodies were obtained from (Sigma–Aldrich, St. Louis, MO, USA).

Densitometric analysis was performed using Quantity One Analysis Software (Bio-Rad Laboratories, Hercules, CA, USA).

### 4.5. Progesterone Level Determination

Progesterone concentration in serum was measured using the RIA method, using a commercial immunoassay kit according to the manufacturer’s instructions (Institute of Atomic Energy, POLATOM, Radioisotope Centre, Poland). Briefly, the progesterone RIA method is based on a competitive binding assay. It involves the competition between unlabeled progesterone in the sample and a fixed amount of ^125^I-labeled progesterone (tracer) for limited binding sites on progesterone-specific monoclonal antibodies. The immunocomplex is immobilized on the reactive surfaces of the test tubes (the tubes are coated with streptavidin so that biotinylated antibodies may be incorporated and immobilized). After incubation, an unbound tracer is washed away, and the bound ^125^I-progesterone is measured using a gamma counter. A standard curve is created based on the values obtained for standards with known progesterone concentrations. The level of progesterone in unknown samples is determined by interpolation into this curve. The antigen concentration is inversely proportional to the measured radioactivity.

### 4.6. Statistics

The statistical significance of differences between the groups was assessed by one-way analysis of variance (ANOVA), and Tukey’s post hoc test was used to further determine the differences’ significance. The SigmaStat 3.0 software (Sigma Stat Inc., San Jose, CA, USA) was used. Differences between the groups were considered significant when *p* < 0.05. Pearson’s correlation coefficient was calculated to assess the correlation between progesterone concentration and relative MTs mRNA level. All data are presented as means of values (*n* = 10) ± standard error of the mean (±SEM).

## Figures and Tables

**Table 1:** Daily food intake by control and progesterone-treated rats. The average amounts of food consumed per 24 h ± SEM of ten animals. * *p* < 0.05 control female rats versus progesterone-treated female rats; ns—non-significant.

Gender	Control	Progesterone-Treated	Statistical Significance
Female	19 ± 0.2 g	21 ± 0.4 g	*p* < 0.05 *
Male	27 ± 0.5 g	26 ± 0.4 g	ns

## Data Availability

The data obtained are presented in the article, and further inquiries can be directed to the corresponding authors: ewa.stelmanska@gumed.edu.pl; szrok@gumed.edu.pl.

## Supplementary Materials

The supporting information can be downloaded at: https://www.mdpi.com/article/10.3390/ijms26094066/s1.

## Abbreviations

The following abbreviations are used in this manuscript:

BAT	Brown adipose tissue
MT	Metallothionein
PCR	Polymerase chain reaction
WAT	White adipose tissue
WATi	Inguinal white adipose tissue
