# Supplementary material for: Gender-Dependent Effect of Progesterone on the Expression of Metallothionein Genes in Rat Inguinal Adipose Tissue
Source: Int J Mol Sci. 2025 Apr 25;26(9):4066. doi: 10.3390/ijms26094066 (PMC12071332; doi:10.3390/ijms26094066)
Supplement: Supplementary file 1 [file ijms-26-04066-s001.zip › ijms-3558871-supplementary.pdf]

Table S1

Composition of laboratory diet

Component	Amount
**Energy (kcal/kg)**	3,100
**Nutrients (mean content in the diet, %)**	
Crude protein	22.0
Crude fiber	6.0
Crude fat	4.2
Starch	27.0
Ash	5.5
Moisture	13.0
**Minerals (mean content in 1 kg diet, mg)**	
Calcium	10,000
Phosphorus	7,500
Magnesium	2,400
potassium	9,000
Sodium	2,200
Chlorine	2,500
Sulphur	1,900
Iron	250
Manganese	100
Zinc	100
Copper	23
Cobalt	2
Iodine	1
**Vitamins (additive in 1 kg diet)**	
A	15,000 IU
D_3	1,000 IU
E	90.0 mg
K	3.0 mg
B_1	20.0 mg
B_2	16.0 mg
B_6	17.0 mg
B_{12}	80.0 μg
Nicotinic acid	120.0 mg
Folic acid	5.0 mg
Pantothenic acid	30.0 mg
Biotin	0.4 mg
Choline	2700.0 mg
